# Treatment Options for Lipodystrophy in Children

**DOI:** 10.3389/fendo.2022.879979

**Published:** 2022-05-04

**Authors:** Francesca Mainieri, Veronica Maria Tagi, Francesco Chiarelli

**Affiliations:** Department of Paediatrics, University of Chieti, Chieti, Italy

**Keywords:** lipodystrophy, leptin, metreleptin, insulin resistance, metabolic complications, adipose tissue

## Abstract

Lipodystrophy includes a heterogeneous group of rare diseases characterized by different amounts of adipose tissue loss and several metabolic complications, including hypertriglyceridemia, steatohepatitis and particularly insulin resistance, that may lead to severe morbidity and, sometimes, mortality. Therefore, therapy for lipodystrophy primarily consists of a conventional approach that involves standard treatments of metabolic abnormalities. Given the evidence of leptin deficiency in lipodystrophy syndromes, leptin replacement therapy has been considered as a treatment option. Long-term studies on the use of therapy with a methionylated analog of human leptin, metreleptin, first on animals and subsequently on human patients, demonstrated enormous improvements of patients’ clinical features and metabolic conditions. Recently, metreleptin was approved by Food and Drug Administration (FDA) for the treatment of generalized lipodystrophy and by European Medicines Agency (EMA) for the treatment of both generalized and partial lipodystrophy. However, further research is being conducted for new and different therapeutic agents, especially helpful for the treatment of patients with partial lipodystrophy, as some of them do not have access to metreleptin therapy or show poor response.

## 1 Introduction

Lipodystrophies are a cluster of heterogeneous syndromes characterized by various degrees of fat loss and different time for symptom onset in a genetic or acquired etiology. According to the extent and body distribution of adipose tissue loss, they can be divided into generalized, partial and localized forms (1). The selective absence of subcutaneous body fat results in reduced energy storage capacity, incorrect lipid storage principally in muscle and liver, and reduced levels of adipokines, such as leptin (2). However, no pathophysiological rule determining this particular fat distribution has been identified (3). Lipodystrophy is rare and often underestimated, however the estimated prevalence is 1.3-4.7 cases per million (4). Lipodystrophic patients, regardless of their genotypic and phenotypic features, tend to develop insulin resistance (IR), together with the numerous related complications, including diabetes mellitus, elevated triglycerides, fatty liver disease, acanthosis nigricans, hypertension, polycystic ovary syndrome (PCOS) (5). Additionally, lipodystrophies can affect patients’ quality of life and psychological well-being (6). Considering the deep impact on patients’ health, appropriate and safe treatment options are crucial. The aim of this minireview is to summarize the therapeutic strategies for lipodystrophy, showing the standard treatment options and metreleptin therapy.

## 2 Therapeutic Approach

**Figure 1:**
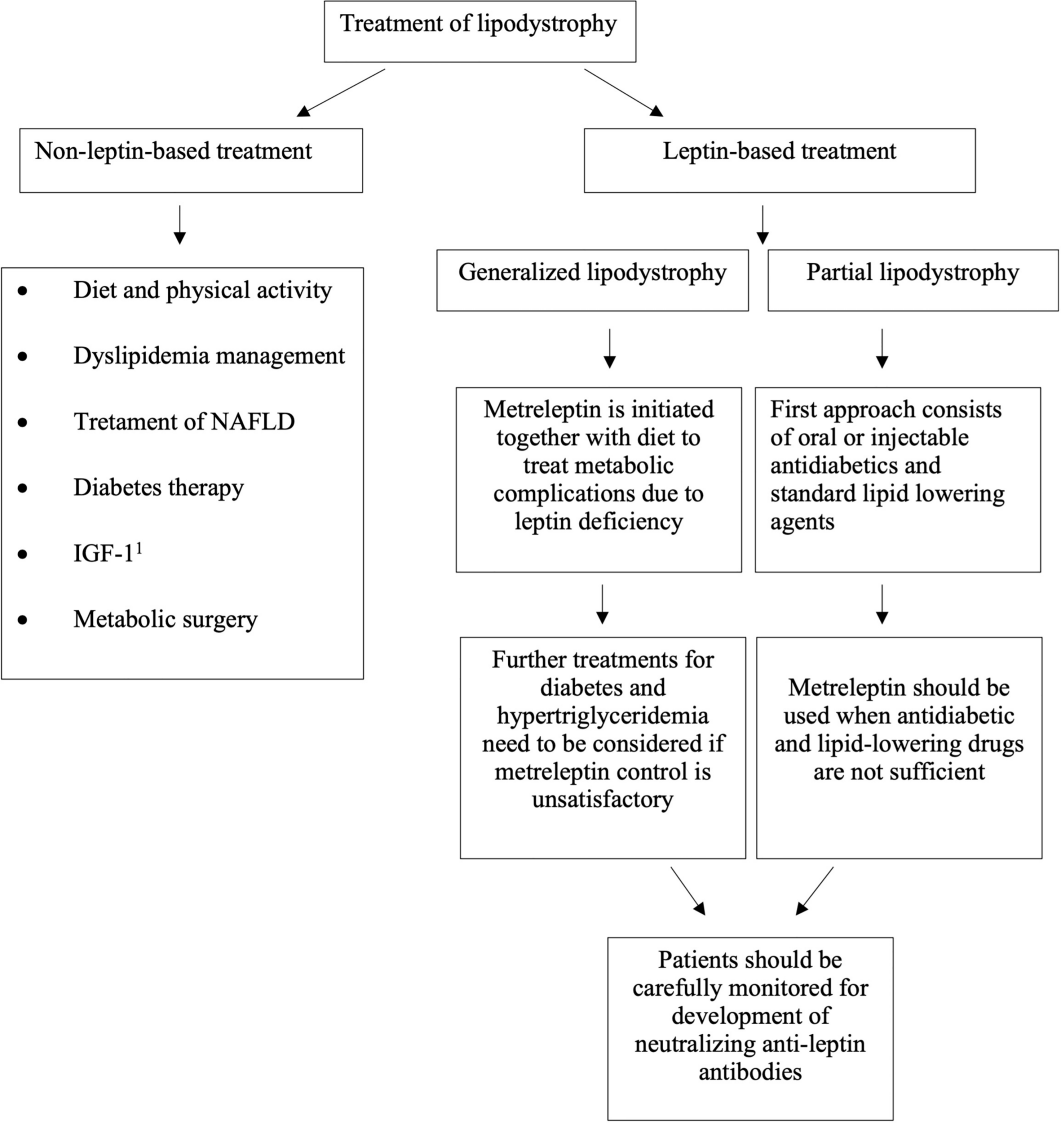
Algorithm for the treatment of patients with lipodystrophy.

Unfortunately, a curative treatment for lipodystrophy has not been identified so far. Consequently, our therapeutic intervention aims to improve the various typically associated diseases. Given the broad spectrum of representative metabolic conditions, specific therapeutic approaches need to be accomplished, as shown in **Figure 1**.

### 2.1 Non-Leptin-Based Treatment

#### 2.1.1 Diet and Physical Activity

Lifestyle intervention reflects the earliest clinical treatment. Practice guidelines recommend a balanced macronutrient diet, consisting of 50-60% carbohydrates, 20-30% fat and 20% protein (6). Patients presenting hypertriglyceridemia and hyperglycemia are both encouraged to follow a low-fat diet, diabetic patients should also consider a reduced carbohydrate intake (7). Fiber and foods rich in omega-3 fatty acids are strongly recommended, while alcohol-based beverages should be avoided (8). However, good adherence to diet is challenging, considering both the leptin deficiency causing hyperphagia and the more likely lower self-control during childhood and adolescence. Over-feeding would be a mistake worsening the metabolic status (9), as would smoking. Selected adult patients show good tolerance of both the combination of phentermine and topiramate (Qsymia), however without solid evidence. Physical activity involving different types of exercise, in view of the hypertrophic fat depots localizations and patients’ contraindications, can be very useful (10). Muscoloskeletal pain, fatigue and psychological stress, typically present, may negatively affect physical performances (6).

#### 2.1.2 Treatment of Dyslipidemia

When lifestyle modifications are not sufficient to control dyslipidemia, general current guidelines need to be used also for lipodystrophic patients. Therefore, statins and fibrates might be used (11). When combined therapy is needed, the risk for myopathy and hepatotoxicity requests to be managed. Although there is no satisfactory evidence for omega-3-fatty acids administration, they are commonly used. Apheresis is an option in case lipid-lowering medications are not effective in patients with severe hypertriglyceridemia unresponsive to other treatments or when they are at risk of acute pancreatitis (6).

Moreover, recommended medications for non-alcoholic fatty liver disease (NAFLD) include metformin and thiazolidinediones (TZD), that demonstrated positive outcomes on liver function and fatty liver disease (12, 13).

#### 2.1.3 Treatment of Diabetes

Diabetes is one of the most frequent metabolic disorders related to lipodystrophy. International or national treatment guidelines are observed also for the majority of lipodystrophic syndromes. To improve severe IR, insulin therapy is often insufficient (14). Additionally, injectable medications might be affected in their kinetics and dynamics by the loss of fat tissue. Metformin is certainly the best drug to reduce IR and improve hyperglycemia. Sulfonylureas or glinides are not recommended in monotherapy because of their ineffectiveness. Recently, the incretin modulators, glucagon-like peptide-1 (GLP-1) receptor agonists or dipeptidyl peptidase-4 (DPP-4) inhibitors, exert glycemic control effects by blocking glucagon secretion. Sodium-glucose cotransporter 2 (SGLT2) inhibitors and alpha-glucosidase inhibitors can apply an insulin-independent action on the regulation of blood glucose levels in lipodystrophy syndromes (15). Additionally, TZD improve glycemic control or hypertriglyceridemia in adult patients with partial lipodystrophy, however often causing augmented ectopic fat and the development of lipomas (16, 17). Nevertheless, evidence of cardiovascular protection in lipodystrophic patients is still poor (18). Angiotensin-converting enzyme (ACE) inhibitors are useful to protect the kidneys and avoid proteinuria (19).

#### 2.1.4 Insulin-Like Growth Factor 1 (IGF-1)

Insulin-like growth factor 1 (IGF-1) at high concentrations is seen to lower blood glucose levels in case of diabetes characterized by severe IR. An explanation could be the similarity of the biochemical structure between insulin and IGF-1 (20). Nonetheless, IGF-1 should not be considered an alternative therapy in lipodystrophy because, by promoting cell growth and differentiation, it might degenerate hypertrophic cardiomyopathy and other typical conditions (10).

#### 2.1.5 Other

Lipodystrophic patients experience a difficult psychological condition due to their physical appearance. The lack of adipose tissue and ectopic fat depots have such an impact on the quality of their lives, causing anxiety and depression. However, this condition can be linked to physical concern, underlying disease mechanisms or both (21). Therefore, patients who aspire to a better cosmetic appearance may consider having cosmetic surgery (22). Excess unwanted localized fat tissue can be surgically excised or removed by liposuction (23). In contrast, lipoatrophic regions may be treated by receiving autologous adipose tissue transplantation, implantation of dermal fillers and facial reconstruction (24). Local injections of deoxycholic acid or cool sculpting technologies are recent alternatives (25, 26).

### 2.2 Leptin-Based Treatment

Lipodystrophy syndromes have been associated for a long time with a poor prognosis, because of the modest results obtained from the antidiabetic and lipid-lowering drugs.

During the last decades, reduced plasma leptin levels were identified as probably related to the pathophysiology of lipodystrophic syndromes. These findings paved the road to leptin replacement therapy (metreleptin therapy), improving the overall prognosis (27).

#### 2.2.1 General Characteristics and Role of Leptin

Dr Jeffrey Friedman discovered leptin, as a function of the adipose tissue mass (28). Adipose tissue synthesizes and secretes leptin and other adipokines, such as adiponectin, all intensely involved in the regulation of glucose and lipid metabolism (29). Leptin is a 16 kilodaltons (kDa) protein consisting of 167 amino-acids and a 4-helix bundle motif (30). Leptin levels are generally higher in women who present a higher percentage of body fat. Leptin deficiency correlates directly with the amount of adipose tissue and potentially determines the development of metabolic abnormalities (31). After being realeased in a pulsatile manner following a circadian rhythm, leptin reaches its receptor typically located in the hypothalamus, where appetite and food intake are regulated (32, 33). In addition, leptin has a crucial metabolic influence predominantly on skeletal muscle, liver, pancreatic islets and adipose tissue (30, 34). In particular, leptin acts to improve insulin action, by increasing glucose uptake and stimulating free fatty acid oxidation in both skeletal muscle and liver, reducing gluconeogenesis and stimulating lipolysis in the liver (35–37). Triacylglycerol and glucose metabolism are other peripheral activities of leptin (38). Specifically, leptin levels are particularly low in generalized lipodystrophy (GL) considering the near total lack of fat tissue, while in partial lipodystrophy (PL) they can be higher or lower, according to the amount of adipose tissue (39, 40). Ultimately, the consequences of leptin deficiency have repercussions on insulin actions as well as on the appetite, determining hyperphagia (41).

#### 2.2.2 The Road to Leptin Replacement Therapy

Studies on animals validated the role of adipose tissue in the pathogenesis of metabolic complications of lipodystrophy (42–44), all supporting and confirming the role of leptin therapy in improving the metabolic parameters. Leptin efficacy shown in mice was then confirmed also in human patients with lipodystrophy (45). Specifically, a small cohort of nine patients with severe lipodystrophy (eight patients with GL and one patient with PL) and baseline serum leptin level of less than 4 ng/ml received the first dose of leptin. An important decrease in HbA1c in eight of the nine patients whit diabetes, an improvement in sensitivity to insulin, reduced triglyceride levels after 4 months of treatment, liver volume decreased, declined levels of liver enzymes and daily caloric intake definitely reduced were registered. As a confirmatory step, the discontinuation of metreleptin therapy caused the increase of fasting triglyceride and insulin levels in the first 48 hours, resolved by resuming therapy (2). Beltrand et al. in 2007 conducted the first open trial where leptin replacement treatment was given daily for 4 months to children with Berardinelli-Seip congenital lipoatrophy and metabolic complications, but without diabetes at a dosage that reached physiological levels. The consequence was the regression of metabolic abnormalities, in particular a reduction of fasting triglycerides level, an increase in insulin sensitivity and a reduction of liver volume (46).

#### 2.2.3 Metreleptin Therapy

Metreleptin is an analog of human leptin made up of 147 amino-acid chain, obtained through recombinant DNA technology and differs from the native human leptin by the addition of a methionyl group at its amino terminus. It is a nonglycosylated polypeptide with one disulfide bond between Cys-97 and Cys-147, with a molecular weight of approximately 16.15 kDa (Myalept website: http://www.myalept.com/pdfs/pi_myalept.pdf). Metreleptin is the first drug of the class of leptin analogs to be approved, designed as an orphan drug in 2001 (47). The first global approval of metreleptin for the treatment of diabetes and/or hypertriglyceridemia in lipodystrophic patients (congenital or acquired, both generalized and partial) was in Japan in March 2013 (48). Up to 2014, at the Clinical Center of the National Institutes of Health (NIH) 55 patients with GL received metreleptin as therapy and this led to the Food and Drug Administration (FDA) approval of metreleptin for the treatment of GL in February 2014 (Food and Drug Administration website: http://www.accessdata.fda.gov/drugsatfda_docs/label/2014/125390s004lbl.pdf). These preliminary results were afterward endorsed by several studies (49–52). On May 31, 2018 the European Medicines Agency’s (EMA) Committee for Medicinal Products for Human Use (CHMP) granted the marketing authorization of metreleptin for patients with GL > 2 years of age and for patients with PL > 12 years of age for whom standard treatments have failed to achieve adequate metabolic control (European Medicines Agency website: https://www.ema.europa.eu/en/medicines/human/EPAR/myalepta). Soon after, on July 31, 2018 there was approval in the European Union and the United Kingdom. In **Figure 1** is shown the algorithm for metreleptin treatment. Instead, metreleptin is not approved as therapy in patients with HIV-related lipodystrophy. In Europe, Northern Africa, the Middle East etc, metreleptin is available in compassionate use programs.

When metabolic conditions are detected in a lipodystrophic patient and conventional therapies are not satisfactory, leptin replacement therapy is authorized (19). According to FDA data, the recommended starting dose of metreleptin is 5 mg/day in women greater than 40 kg, 2.5 mg/day in men greater than 40 kg, and 0.06 mg/kg/day (0.012 ml/kg) in women and men less than or equal to 40 kg. In children, the dose should be based on their weight in kilograms, thus requiring higher doses particularly during puberty (8). Metreleptin administration is once a day at the same time every day, subcutaneously, but because of its short half-life (3.8-4.7 hours), sometimes the dose is divided into two subcutaneous injections. Renal clearance is thought to be the major route of elimination (53).

#### 2.2.4 Beneficial Effects

Especially in pediatric patients with lipodystrophy, metreleptin effects are notable, as demonstrated by Brown et al. (54). This study showed significant improvement in metabolic abnormalities linked to lipodystrophy and low baseline leptin levels, registered both in the short (1 year) and long term (mean, 5 years), with the overall result of patients’ quality of life amelioration. Diabetic patients obtained a reduction in HbA1c levels and microvascular complications. Given the improvements of metreleptin in insulin sensitivity (55, 56), half of the patients obtained the discontinuation of insulin therapy after 1-year administration of metreleptin. A triglyceride blood levels reduction was observed in more than one-quarter of patients in therapy for at least 1 year, as well as reduced hepatocellular injury and liver enzyme levels. The best outcomes were registered in adolescents, who showed more severe metabolic involvement before the treatment. So, children need to be treated with metreleptin in order to avoid metabolic degeneration, mostly during puberty (57). Metreleptin treatment has also shown to classically improve hepatic steatosis (58). As leptin exerts a key role in reproduction (59), consequently metreleptin treatment avoids the progression of delayed puberty, without causing precocious puberty. Further positive effects of metreleptin include control of gonadotropin secretion (60), protection of the kidneys and decreased proteinuria (61), reinforcement in immunoregulation (62), and reduction in androgen levels in the context of PCOS (63).

#### 2.2.5 Response to Metreleptin and Dose Adjustment

Patients with GL achieve significant outcomes thanks to metreleptin therapy, as indicated in **Table 1**. Conversely, in PL patients the response to metreleptin treatment is less solid (51, 64, 65). Generally, patients with PL showing moderately to severely low leptin and important baseline metabolic disorders tend to benefit more from metreleptin (51, 66).

**Table 1:** Major Effects of Metreleptin Treatment in Generalized Lipodystrophy.

Clinical Conditions	Major Metreleptin Effects
Hyperphagia	Decreased, leading to reduced appetite
Insulin Resistance and Diabetes	Reduced, leading to improvement of fasting glucose, reduced HbA1c, decreased insulin doses or discontinuation of insulin therapy
Dyslipidemia	Lowering of triglycerides and preventing acute pancreatitis
Liver disorders	Decreased hepatic steatosis and serum transaminases, avoiding the development of steatohepatitis
Kidney involvement	Reduced hyperfiltration and proteinuria
Reproductive System	Normal gonadotropin secretion, leading to normal progression of puberty, normalized menstrual cycles and improved fertility. In women, decreased testosterone, while in males increased testosterone

The first metabolic changes appear within 4-6 weeks from the initiation of therapy: reduced appetite is one of the earliest. According to NIH awareness, if body weight decrease is more than 10% in a patient, a dose adjustment is needed, still using the total mg/kg/day dosage, that instead will be decreased in case of further weight loss 1 month after the adjustment. Every dose adjustment must be maintained for at least 4 weeks and metabolic progression must be observed (19). Definitely, metreleptin doses should be adjusted every 3-6 months according to tolerability issues, metabolic parameters and weight change (22). Occasionally, a reduced metreleptin efficacy could derive from a non- adherence to the prescribed regimen. Alternatives may be using very short needles for the injection and more management collaboration with patients (19).

#### 2.2.6 Quality of Life and Survival

Cook et al. (67) assessed the overall quality-of-life impact of lipodystrophy and the benefits associated with metreleptin, and more than half of all patients perceived great improvement in their metabolic disorders. Hyperphagia, inability to work/attend school, pancreatitis, elevated transaminases, hyperglycemia and hypertriglyceridemia registered a higher decrease in prevalence. Generally, patients with GL and PL experienced a poor quality of life, but metreleptin treatment has shown to reduce the gap in quality of life between untreated GL/PL and perfect healthy status, by around 59% and 31%, respectively. Similar results have not been reported in patients affected by chronic pathologies (68).

In another study, Cook et al. (69) presented a lower risk of mortality for a mixed cohort of GL and PL patients treated with metreleptin than metreleptin-naïve patients. The improvement of metabolic complications through metreleptin therapy may be linked to better survival.

#### 2.2.7 Adverse Effects

Side effects provoked by metreleptin treatment are quite rare and generally mild to moderate (70). The most frequent adverse effect is injection site reaction (bruising and urticaria), followed by headache (13%), hypoglycemia (13%), decreased weight (13%) and abdominal pain (10%) (71). Urticaria, anaphylaxis, papular rash, angioedema, and pruritus are immune-related hypersensitivity reactions also reported (https://www.accessdata.fda.gov/drugsatfda_docs/label/2014/125390s004lbl.pdf). Instead, the more severe side effects, although rare, are lymphoproliferative disorders, anti-leptin antibodies, pancreatitis, hypoglycemia, autoimmunity and immune-related hypersensitivity. Hypoglycemia can be the consequence of improved insulin action due to metreleptin treatment, usually seen in diabetic patients in simultaneous therapy with insulin or oral hypoglycemic drugs (70). Natural history of some lipodystrophy syndromes may be characterized by lymphoproliferative disorders. Thus, it is not likely to have a direct cause and effect relationship between metreleptin therapy and development of lymphomas (72, 73). However, three patients with acquired generalized lipodystrophy (AGL) in therapy with metreleptin have been affected by T cell lymphoma, two of them presented with immunodeficiency and bone marrow failure before treatment (74). So, when prescribing metreleptin treatment, it is good practice to consider the possible presence of hematologic abnormalities. Although with still poor evidence, leptin replacement has been associated with the pathogenesis of obesity-associated cancers (e.g. breast and colon cancer) (75). While on metreleptin therapy, in a substantial number of patients anti-leptin antibodies are produced, some of them with neutralizing action, compromising the efficacy of both leptin and metreleptin (76). Worsened metabolic control and damage of immune function, determining severe and more frequent infections, might be potential sequelae. Metreleptin’s black box warnings are concerns for lymphoproliferative disorders and the production of anti-leptin antibodies (47). Acute pancreatitis, can occur also when tapering or acutely interrupting metreleptin therapy (57, 70). Autoimmune hepatitis and membranoproliferative glomerulonephritis are also reported, considering metreleptin impact on autoimmunity (49). Occasionally, the same disorders are seen before initiating metreleptin treatment, especially in patients with baseline fatty liver or renal disease secondary to lipodystrophy itself (77).

## 3 Other Therapeutic Strategies

Whether the success of metreleptin therapy has been widely demonstrated in patients with GL, unfortunately the same results have not been detected in all patients with PL (6). Given that therapy with metreleptin is not accessible to the great part of patients with PL or a lack of response is registered, new therapeutic strategies are currently being investigated. Specifically, liver-specific treatment strategies, agents working on hepatic lipid metabolism and other medications are under development for patients with PL forms (78).

## 4 Conclusion

Lipodystrophy syndromes are characterized by various and severe metabolic complications, treatment of which is challenging. Since standard therapies often realized an insufficient metabolic regulation, leptin replacement therapy with metreleptin, a recombinant leptin analog, is needed. Metreleptin therapy has been approved by FDA and EMA, respectively for patients with GL and for patients with both GL and PL. Patients with GL commonly benefit from therapy with metreleptin, that is generally used as an adjunct to diet, physical activity and other standard treatments for metabolic abnormalities. For a subgroup of patients with PL, metreleptin may be a suitable treatment option, while for others it might not represent an adequate therapy. For these patients, additional studies on metabolic pathways are necessary to develop novel therapeutic strategies.

## Author Contributions

FM conceptualized the study, drafted the initial manuscript, helped by VT in revising the manuscript. FC supervised and critically reviewed the manuscript for important intellectual content. All authors agree to be accountable for the content of the work. All authors contributed to the article and approved the submitted version.

## Funding

Funding for publication has been received from the University of Chieti, Chieti, Italy.

## Conflict of Interest

The authors declare that the research was conducted in the absence of any commercial or financial relationships that could be construed as a potential conflict of interest.

## Publisher’s Note

All claims expressed in this article are solely those of the authors and do not necessarily represent those of their affiliated organizations, or those of the publisher, the editors and the reviewers. Any product that may be evaluated in this article, or claim that may be made by its manufacturer, is not guaranteed or endorsed by the publisher.

## Abbreviations

IR, Insulin resistance; PCOS, polycystic ovary syndrome; NAFLD, non-alcoholic fatty liver disease; TZD, thiazolidinediones; NASH, non-alcoholic steatohepatitis; GLP-1, glucagon-like peptide-1; DPP-4, dipeptidyl peptidase-4; SGLT2, sodium-glucose cotransporter 2; GL, generalized lipodystrophy; PL, partial lipodystrophy; NIH, National Institutes of Health; FDA, Food and Drug Administration; EMA, European Medicines Agency; CHMP, Committee for Medicinal Products for Human Use; CGL, congenital generalized lipodystrophy; AGL, acquired generalized lipodystrophy; FPL, familial partial lipodystrophy.
